# Influence of Disulfide Connectivity on Structure and Bioactivity of α-Conotoxin TxIA

**DOI:** 10.3390/molecules19010966

**Published:** 2014-01-15

**Authors:** Yong Wu, Xiaosa Wu, Jinpeng Yu, Xiaopeng Zhu, Dongting Zhangsun, Sulan Luo

**Affiliations:** Key Laboratory of Tropical Biological Resources, Ministry of Education, Key Lab for Marine Drug of Haikou, Hainan University, Haikou, Hainan 570228, China; E-Mails: wys211@163.com (Y.W.); 7776820@163.com (X.W.); mtying0001@126.com (J.Y.); biozxp@163.com (X.Z.)

**Keywords:** α-conotoxinTxIA, α3β2 nAChRs, disulfide isomerisation, peptide synthesis, oxidative folding

## Abstract

Cone snails express a sophisticated arsenal of small bioactive peptides known as conopeptides or conotoxins (CTxs). Through evolutionary selection, these peptides have gained the ability to interact with a range of ion channels and receptors, such as nicotinic acetylcholine receptors (nAChRs). Here, we used reversed-phase high performance liquid chromatography (RP-HPLC) and electrospray ionization-mass spectrometry (ESI-MS) to explore the venom peptide diversity of *Conus textile*, a species of cone snail native to Hainan, China. One fraction of *C. textile* crude venom potently blocked α3β2 nAChRs. Subsequent purification, synthesis, and tandem mass spectrometric analysis demonstrated that the most active compound in this fraction was identical to α-CTx TxIA, an antagonist of α3β2 nAChRs. Then three disulfide isoforms of α-CTx TxIA were synthesized and their activities were investigated systematically for the first time. As we observed, disulfide isomerisation was particularly important for α-CTx TxIA potency. Although both globular and ribbon isomers showed similar retention times in RP-HPLC, globular TxIA potently inhibited α3β2 nAChRs with an IC_50_ of 5.4 nM, while ribbon TxIA had an IC_50_ of 430 nM. In contrast, beads isomer had little activity towards α3β2 nAChRs. Two-step oxidation synthesis produced the highest yield of α-CTx TxIA native globular isomer, while a one-step production process based on random oxidation folding was not suitable. In summary, this study demonstrated the relationship between conotoxin activity and disulfide connectivity on α-CTx TxIA.

## 1. Introduction

Cone snail venoms represent a vast untapped reservoir of natural products that have potential medicinal value for pharmaceutical development. Various species of marine cone shells have been estimated to produce over 50,000 distinct neurologically active peptides [1,2]. Analysis of these natural toxins may help identify model compounds for the discovery of novel therapeutic agents [3,4,5]. These toxins gain the ability to interact with various ion channels and receptors, such as nicotinic acetylcholine receptors (nAChRs) [3]. The nAChRs are pentameric membrane-bound proteins that are involved in normal physiologic processes and in a wide range of disease states including, pain, addiction, myasthenia gravis, schizophrenia, epilepsy, Alzheimer’s disease, breast and lung carcinoma [6,7].

In the present study, we examined venom peptidome profile from a *C. textile* species native to Hainan, China. We used fractionation followed by ESI-MS in order to isolate and identify novel conotoxins. Each fraction was screened for activity against α3β2 nAChRs. Several fraction components were able to inhibit α3β2 nAChRs. The compound with same molecular mass as the known antagonist of α3β2 nAChRs, α-CTx TxIA, showed highest potency [8].

**Figure 1:**
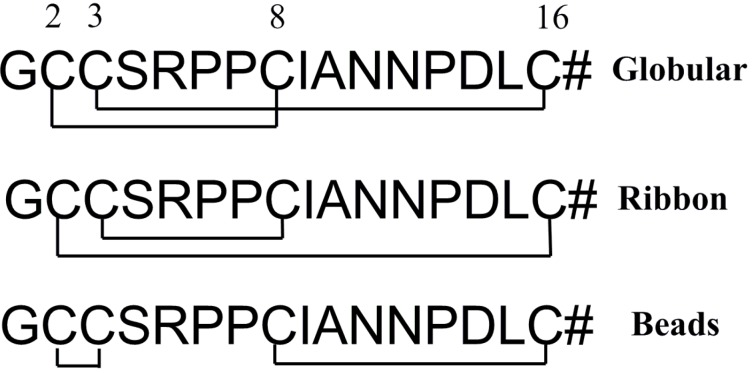
A schematic representation of globular (native), ribbon and beads isomers’ disulfide connectivities of α-CTx TxIA. #, amidated COOH terminal.

α-CTx TxIA is a 16-residue conotoxin with 4/7 intercysteine spacing [8,9]. Previous study reported that TxIA binded with high affinity to AChBPs from different species and selectively targeted the α3β2 nAChRs [8]. Native α-CTx TxIA has two disulfide bridges (Cys2–Cys8 and Cys3–Cys16), which give the molecule a two-loop configuration referred to as the “globular” isomer (Figure 1). Alternative connectivities (Cys2–Cys16 and Cys3–Cys8) and (Cys2–Cys3 and Cys8–Cys18) are referred to as the “ribbon” and “beads” isomers, respectively (Figure 1).

Due to the relative ease of synthesis and small number of possible disulfide bond isomers, α-CTx TxIA presents an ideal model for the study of disulfide bonding effect on peptide structure and function. Hence we synthesized three isomers of α-CTx TxIA with selective oxidation strategy and examined the structures and activities of the globular isomer and non-native isomers. We also compared isomer yields from two-step and one-step oxidation synthesis methods.

## 2. Results and Discussion

### 2.1. Results

#### 2.1.1. Purification and Identification of α-CTx TxIA from *C. textile*

**Figure 2:**
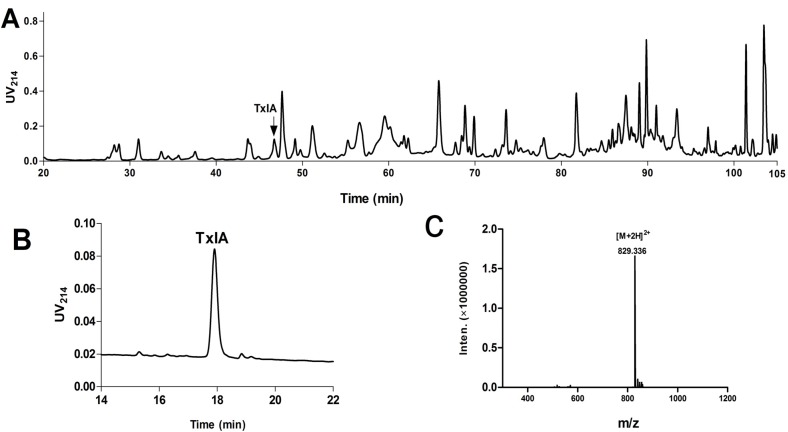
RP-HPLC and ESI-MS analysis of α-CTx TxIA. (**A**) HPLC chromatography of Hainan *C. textile* crude venom using Vydac C18 semi-preparative column (10 μm, 10 mm × 250 mm). The arrow denotes α-CTx TxIA. The linear gradient was a 5%–35% solvent B gradient in 75 min, then 35%–65% gradient in 35 min. (**B**) Analytical HPLC profile of native α-CTx TxIA from *C. textile* venom using a linear gradient of a 10%–30% eluate B, and 90%–70% eluate A over 30 min, In panel A & B, eluate B is 0.05% TFA in 90% ACN, remainder water; eluate A is 0.075% TFA in water. Absorbance was monitored at 214 nm; (**C**) ESI-MS analysis of native α-CTx TxIA with calculated mass of 1,656.67 Da.

**Figure 3:**
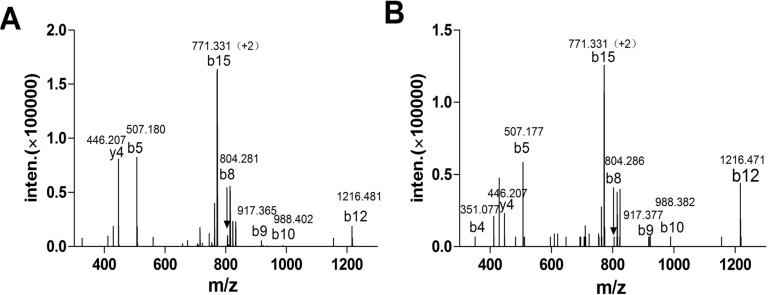
(**A**) Reduced native TxIA MS/MS spectrum of the precursor ion of *m/z* 831.356 [M+2H]^2+^ with the assignment of a series of *b*-ions obtained under collision-induced dissociation (CID) conditions; (**B**) Synthetic linear TxIA MS/MS spectrum of the precursor ion *m/z* 831.356 [M+2H]^2+^, obtained under CID conditions.

The crude venom extracted from Hainan *C. textile* was fractionated by RP-HPLC and more than 90 peaks were detected (Figure 2A). Ninety fractions were collected every minute during the gradient of the 20–110 min for further mass spectrometry analysis. A total of 105 distinct peptide components were detected ranging from 944.40 Da to 4,930.73 Da, with the highest frequency between 1,500~2,000 Da (data not shown). Fractionated venom samples from *C. textile* were tested for activity against rat α3β2 nAChRs heterologously expressed in *Xenopus laevis* oocytes. Several fractions blocked α3β2 nAChRs. We isolated and purified the active peptide from the peak fraction that had highest potency (Figure 2A,B). Monoisotopic molecular weight of this peptide was 1,656.67 Da (Figure 2C), consistent with previously identified α-CTx TxIA. We hypothesized that the peptide isolated from *C. textile* crude venom might be α-CTx TxIA. To confirm this hypothesis, we synthesized globular isomer of α-CTx TxIA with a disulfide connectivity of Cys2–Cys8 and Cys3–Cys16. A co-injection experiment was performed with native peptide and synthetic α-CTx TxIA, which showed identical elution profile. Furthermore, electrospray tandem mass spectrometry (ESI-MSMS) was applied to characterize reduced native peptide and synthetic linear α-CTx TxIA. The native peptide was reduced with dithiothreitol (DTT), then purified and submitted to mass spectrometric analysis. The MS/MS spectrum of reduced native peptide is shown in Figure 3A. The spectrum revealed the presence of ions of *m/z* 507.180 (b4), 804.281 (b8), 917.365 (b9), 988.402 (b10), 1216.481 (b12), 771.331 (b15), 446.207 (y4), which were consistent with theoretical fragment ions of reduced α-CTx TxIA. Figure 3B shows the MS/MS spectrum of synthetic linear α-CTx TxIA, which is similar to the reduced native spectrum. These results confirmed that the most potent component was α-CTx TxIA, an antagonist of α3β2 nAChRs.

#### 2.1.2. Synthesis of α-CTx TxIA Isomers by Two-Step Oxidation

**Figure 4:**
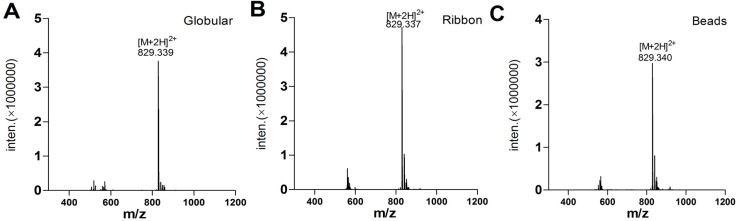
Three synthetic α-CTx TxIA isomers’ mass analyzed by ESI-MS. (**A**) Globular isomer; (**B**) Ribbon isomer; (**C**) Beads isomer.

**Figure 5:**
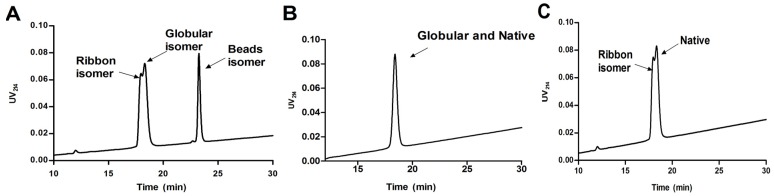
HPLC analysis of native and synthetic α-CTx TxIA isomers co-injection. (**A**) Co-injection traces of three α-CTx TxIA isomers synthesized by two-step oxidation method. (**B**) Co-injection traces of synthetic globular TxIA and native peptide. (**C**) Co-injection traces of synthetic ribbon and native TxIA. Peptides were analyzed on a reversed-phase analytical Vydac C*18* (5 μm, 4.6 mm × 250 mm) HPLC column using a linear gradient of a 10%–30% eluate B, and 90%–70% eluate A over 30 min, where B = 0.05% TFA in 90% ACN, remainder water; A = 0.075% TFA in water. Absorbance was monitored at 214 nm.

In order to determine the effect of α-CTx TxIA disulfide connectivity on biological activity, three possible isomers (globular, ribbon, and beads, Figure 1) were synthesized by a regioselective two-step oxidation method. Three linear peptides were synthesized using the Fmoc protocols described in the Experimental. Peptide was cleaved from the resin together with all *S*-Acm but not the other protecting groups. Next, free cysteine residues were oxidized using potassium ferricyanide to form the first disulfide bond. After HPLC purification of monocyclic peptide, iodine treatment was used to form the second disulfide in the intermediate containing *S-*Acm protecting groups. Folded peptide isomers were individually purified by HPLC. Electrospray mass spectrometry was utilized to confirm the identity of synthetic TxIA isomers, and the results showed they had same monoisotopic molecular mass (Figure 4). HPLC analysis of a mixture of three synthetic isomers showed globular and ribbon isomer had similar hydrophilicity (Figure 5A), whereas beads isomer was more hydrophobic with later retention time than the other isomers. HPLC analysis of a mixture of either ribbon or globular isomer with native toxin indicated that native peptide co-eluted with globular isomer but not with ribbon isomer (Figure 5B,C). This result demonstrated that native peptide had Cys2-Cys8 and Cys3-Cys16 connectivity, which was typical for globular isomer.

#### 2.1.3. Peptide Synthesis of α-CTx TxIA by One-Step Oxidation

**Figure 6:**
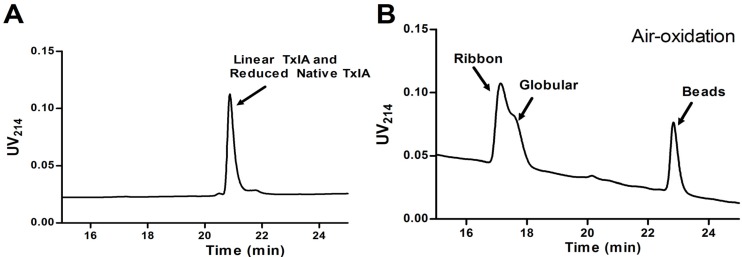
Air oxidation random folding of α-CTx TxIA linear peptide in 0.1 M NH_4_HCO_3_ buffer. (**A**) Co-injection trace of synthetic linear peptide and reduced native α-CTx TxIA; (**B**) RP-HPLC analysis after random folding. Peptides were analyzed on a reversed phase analytical Vydac C_18_ (5 μm, 4.6 mm × 250 mm) HPLC column using a linear gradient of a 10%–30% eluate B, and 90%–70% eluate A over 30 min, where B = 0.05% TFA in 90% ACN; A = 0.075% TFA in water. Absorbance was monitored at 214 nm.

Generally, α-CTx synthesis by a two-step oxidation method is more complicated and expensive than one-step oxidation. In order to explore simpler synthesis methods, we synthesized α-CTx TxIA by a one-step oxidation method. One-step air oxidation folding of full reduced native and synthesized linear peptide of α-CTx TxIA was conducted in regular 0.1 M NH_4_HCO_3_ (pH 8.0, RT). Irrespective of the starting material, each folding reaction resulted in three products corresponding to three isomers of α-CTx TxIA (Figure 6B). RP-HPLC indicated that this folding method produced a greater amount of ribbon isomer, whereas globular and beads isomers were formed in similar amounts. However, globular and ribbon isomers were not well resolved by HPLC. Fully reduced native α-CTx TxIA and linear synthetic peptide eluted at the same retention time (Figure 6A), and reduced native α-CTx TxIA after refolding showed a similar HPLC profile as the peptide synthesized by one-step oxidation (Figure 6B). These results indicated that two-step oxidation synthesis was a much better method than one-step oxidation for α-CTx TxIA globular (native) isomer with a disulfide bond connectivity linking the 1st Cys to the 3rd Cys and the 2nd Cys to the 4th Cys. So one-step oxidation synthesis by random folding was not suitable for synthesizing active α-CTx TxIA.

#### 2.1.4. α-CTx TxIA Isomers Exhibit Differential Activity Against α3β2 nAChRs

**Figure 7:**
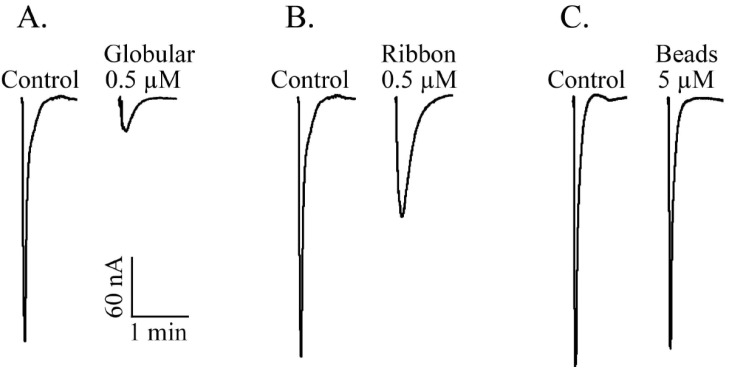
Representative ACh-evoked currents of rat α3β2 nAChRs expressed in *Xenopus* oocytes obtained in the absence (Control) and presence of 3 isomers of α-CTx TxIA. (**A**) Globular, (**B**) Ribbon, and (**C**) Beads.

**Figure 8:**
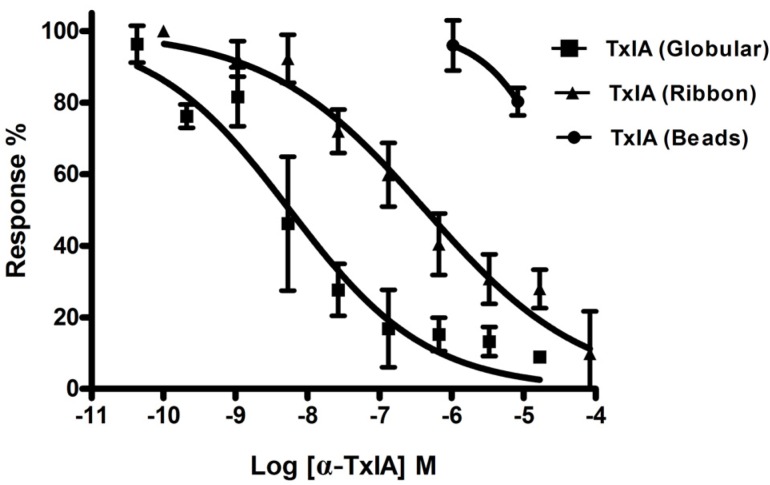
Inhibition concentration-response curves for the isomers of α-CTx TxIA. Values are mean ± SEM from 6 to 10 separate oocytes. Globular, Ribbon and Beads isomers were tested on rat α3β2 nAChRs expressed in *Xenopus* oocytes.

To compare the activity of the three disulfide isomers of α-CTx TxIA synthesized by two-step oxidation, the bioactivity of each isomer was individually examined in an electrophysiology assay. This method measured the inhibition effect of each isomer on rat α3β2 nAChRs expressed in *Xenopus* oocytes. Figure 7 shows representative responses to ACh of α3β2 nAChRs in the presence and absence of each isomer. Globular TxIA at 0.5 μM concentration produced an almost complete inhibition of α3β2 nAChRs (Figure 7A). At the same concentration, ribbon TxIA exhibited ~65% inhibition of ACh evoked current amplitude produced by α3β2 nAChRs (Figure 7B), and the beads isomer failed to inhibit the response at high concentration (5 μM) (Figure 7C). We tested all three isomers at a variety of concentrations on α3β2 nAChRs, and determined the degree of inhibition for each concentration (Figure 8). Globular TxIA reversibly inhibited ACh-evoked currents mediated by α3β2 nAChRs with an IC_50_ of 5.4 nM, whereas ribbon TxIA was characterized by an IC_50_ of 430 nM on α3β2 nAChRs. The native globular isomer was ~80-fold more potent than ribbon isomer. The beads isomer had little activity on α3β2 nAChRs (Figure 8). Therefore, disulfide isomerisation was particularly important for α-CTx TxIA potency. The native globular TxIA was the most potent isoform.

#### 2.1.5. Circular Dichroism (CD) Analysis of α-CTx TxIA Isomers

**Figure 9:**
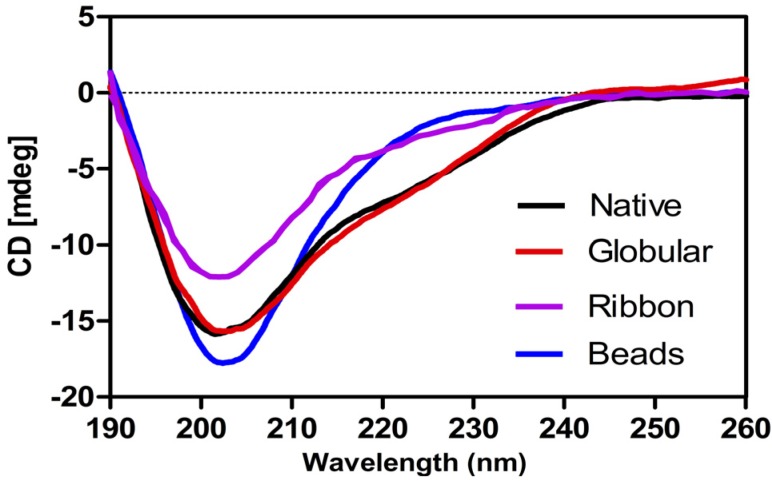
Circular dichroism (CD) spectra of native α-CTx TxIA and its isomers.

Secondary structures of three synthetic isomers and native α-CTx TxIA were analyzed by CD spectroscopy (Figure 9). Globular isomer and native TxIA contained some α-helical structure, as indicated by a double minimum around 208 and 222 nm. This result was consistent with previous structural studies of α-conotoxins [10]. On the other hand, beads isomer exhibited a minimum at around 200 nm, indicative of a random coil conformation with no α-helical and β-sheet, and ribbon isomer was between them. These secondary structure differences likely result in a weaker potency of ribbon isomer and little activity of the beads isomer. Available literature data indicated that disulfide linkages significantly change α-conotoxins’ secondary structures [11,12]. Generally, a disulfide bond between adjacent cysteines is energetically unfavorable [13,14]. Formation of the correct disulfide bond is important in maintaining structure and activity for α-CTx TxIA.

### 2.2. Discussion

Cone snail venom is composed of a complex mixture of different bioactive peptides [15,16]. By optimized liquid chromatography and electrospray ionization mass spectrometry method, 105 obvious components were identified in Hainan *C. textile* crude venom. Monoisotopic molecular masses of fractionated conotoxins were manually compared to the masses of known wild type *C. textile* conopeptides listed in Conoserver, a database for conopeptide sequences and structures [17,18]. Among 105 peptides from Hainan *C. textile* venom, 90% exhibited different masses, while 11 conotoxins showed the same mass as previous research. We also found that conotoxins from *C. textile* of Hainan, China exhibited significant differences compared to other geographic species. In order to further explore venom peptide complexity and diversity, we are planning future studies to identify venom peptide sequences by MS/MS analysis or Edman degradation.

Different nAChR subtypes are implicated in learning, pain sensation, and neurological diseases such as pain, Parkinson’s disease and nicotine addiction, *etc*. [19,20]. In this study, we used α3β2 nAChRs as a target to identify novel α-conotoxins from *C. textile* venom. The most potent component on α3β2 nAChRs was identified as α-CTx TxIA, which was first discovered by Dutertre *et al.* [8]. Their study showed α-CTx TxIA bonded with high affinity to AChBPs from different species and selectively targeted the α3β2 nAChRs. Structure-activity relationships between TxIA analogs indicated an important role for Arg5 in the high-affinity binding of TxIA to AChBPs [8], but this study did not give more details about the relationship between disulfide connectivity and bioactivity. To address this important question, we used chemical methods to synthesize α-CTx TxIA and its possible isomers by two-step and one-step oxidation methods. In order to control disulfide bond formation, we chose a selective oxidation strategy for the two-step oxidation synthesis of α-CTx TxIA using *S-*Trt in combination with the *S-*Acm protecting group by Fmoc chemistry. The two-step oxidation method was superior to one-step oxidation synthesis, because globular and ribbon isomers were overlapped and not well resolved in the HPLC profile. Therefore synthetic peptide isomers with different disulfide bond arrangements produced by two-step oxidation were used for all further analysis.

Prior studies have indicated that the globular conformation of α-conotoxins is the most stable. Formation of ribbon and beads isomers of α-conotoxins induces structural distortions and decreases conformational stability that leads to lower biological activity compared with the globular isomer. Pharmacological studies have traditionally focused on the activities of the globular form, assuming this form to be the native disulfide connectivity. However, one study reported that the α-CTx AuIB ribbon isomer could be up to 10 times more potent than native peptide [21]. In another finding non-native ribbon α-CTx BuIA isomer possessed a single well-defined conformation [11]. Moreover, the related χ/λ-conotoxins exhibit the ribbon disulfide topology as their native form [22,23]. These findings together with sequence similarities between α-CTx TxIA and α-CTx AuIB highlight the importance of structure-activity relation studies for different disulfide bond isomers.

In this study, globular isomer strongly inhibited α3β2 nAChRs, whereas ribbon isomer of α-CTx TxIA was ~80 times less potent than the globular one. This result also correlated with the circular dichroism (CD) data showing a decrease in folded conformation in the order: globular isomer > ribbon isomer > beads isomer. CD analysis suggested that non-native peptides likely fail to adopt an appropriate conformation to block α3β2 nAChRs. Of the many published α-conotoxin sequences, several non-native disulfide isomers have also been reported [11,12,21,24]. These studies indicate that alternative disulfide connectivities lead to very different structures and hence to different activities.

## 3. Experimental

### 3.1. Materials

Acetylcholine chloride, atropine, and bovine serum albumin were obtained from Sigma (St. Louis, MO, USA). Reversed-phase HPLC analytical Vydac C18 (5 μm, 4.6 mm × 250 mm) and preparative C_18_ Vydac columns (10 μm, 22 mm × 250 mm) were obtained from Grace Vydac (Hesperia, CA, USA). Reagents for peptide synthesis were from Applied Biosystems (Foster City, CA, USA) and GL Biochem (Shanghai, China). Acetonitrile (HPLC grade) was purchased from Fisher Scientific (Pittsburg, PA, USA). Trifluoroacetic acid was from Tedia Company (Fairfield, OH, USA). All other chemicals used were of analytical grade. Clones of rat α3 and β2 cDNAs were kindly provided by S. Heinemann (Salk Institute, San Diego, CA, USA).

### 3.2. Venom Fractionation

Specimens of *C. textile* were collected from Hainan near the South China Sea. Venom ducts dissected from specimens of *C. textile* were homogenized in 30% acetonitrile/water acidified with 0.1% trifluoracetic acid at 4 °C. Whole extracts were centrifuged at 10,000 *×g* for 15 min, at 4 °C. The supernatants containing the soluble peptides were pooled, lyophilized, and stored at −70 °C for subsequent HPLC separation. The crude venom was dissolved in 0.5% trifluoroacetic acid and injected into the Waters e2695 HPLC system and separated on a Vydac C_18_ column (0.46 cm × 25 cm, 5 μm particle size, 300 Å pore size). The peptides eluted with a flow rate of 1 mL/min using a linear gradient from 95% eluate A, 5% eluate B to 65% eluate A, 35% eluate B in 75 min, then increased to 35% eluate A, 65% eluate B in 35 min. The fractions were manually collected, then lyophilized for ESI-MS and electrophysiology analysis.

### 3.3. Tandem Mass Spectrometric Analysis

MS/MS analyses were conducted on an IT-TOF/MS (Shimadzu, Kyoto, Japan) equipped with an electrospray ionization source. The instrument was set to permit the accumulation of all ions in the octopole, followed by rapid pulsing into the IT for MS/MS analysis, and then ions are introduced into the TOF sector for accurate mass determinations. The setting conditions for operations were: positive mode, CDL temperature 200 °C, block heater temperature 200 °C, nebulizer gas (N_2_) flow of 1.5 L/min, trap cooling gas (Ar) flow of 95 mL/min, electrospray voltage 4.5 kV, ion trap pressure 1.7 × 10^−2^ Pa, TOF region pressure 1.5 × 10^−4^ Pa, ion accumulation time 30 ms, collision energy set at 50% both for MS/MS, and collision gas set to 50%. The mass scale was calibrated externally using a TFA-Na solution. The reduced native and synthetic α-CTx TxIA was injected into mass spectrometer respectively. Data were collected at scan ranges of 500–1,500 for MS and 200–1,500 for MS/MS.

### 3.4. Chemical Synthesis of α-CTx TxIA by Two-Step Oxidation

Resin-bounded peptides of α-CTx TxIA isomers for two-step oxidation were synthesized on an automated peptide synthesizer ABI433 (Applied Biosystems) according to standard FastMoc chemistry as supplied by the manufacturer. Rink amide resin (0.1 mmol) was used as the solid support and yielded amide capping at the *C*-terminus. Side-chain protection of non-Cys residues was in the form of Ser (tBu), Arg(Pbf), Asn(Trt) and Asp(OtBu). Orthogonal protection was used on cysteines: The first disulfide to be formed (2-8, 3-8 or 2-3 for the three isomers, respectively) was protected with Trt groups and the second (3-16, 2-16 or 8-16) with Acm groups. After assembly of the resin-bounded peptide, the terminal Fmoc group was removed *in situ* by treatment with 20% piperidine in *N-*methylpyrrolidone. Resin-bounded peptide cleavage and deprotection was accomplished with a reagent K mixture (82.5% trifluoroacetic acid/5% water/5% phenol/5% thioanisole/2.5% ethanedithiol) for 2 h at room temperature. The crude peptide was precipitated in cold diethyl ether, centrifuged and washed with diethyl ether several times. The linear peptides were purified by reversed-phase HPLC using a preparative C*18* Vydac column with a linear gradient of a 10%–30% eluate B, and 90%–70% eluate A over 30 min. Solvent B was 90% ACN, 0.092% TFA, and H_2_O; Solvent A was 0.1% TFA in H_2_O. The HPLC elution was monitored at 214 nm.

A two-step oxidation protocol was used to fold the peptides selectively, as described previously [17]. Briefly, peptide with the disulfide bridge between Cys2 and Cys8, Cys3 and Cys8, or Cys2 and Cys3, was dissolved in solvent A respectively with final peptide concentration of 50 μM. The peptide solution was added slowly to an equal volume of 20 mM potassium ferricyanide K_3_[Fe(CN)_6_], 0.1 M Tris base, pH adjusted to 7.5 with acetic acid. The solution was mixed to react for 45 min, and the monocyclic peptide was purified by RP-HPLC. Simultaneous removal of the *S*-Acm groups and closure of the disulfide bridge between Cys3 and Cys16, Cys2 and Cys16, Cys8 and Cys16, respectively, was carried out by iodine oxidation. The bicyclic peptide was purified by HPLC on a reversed-phase C18 Vydac column using a linear gradient as above. Identity of the linear peptide and fully folded peptide was confirmed by ESI-MS analysis.

Quantity of peptide was calculated by injecting approximately 1 × 10^−9^ mol of peptide dissolved in 10−20 µL of 0.1% TFA onto an analytical Vydac reversed phase C_18_ column that had a 5 µM particle, 300 Å pore size and was 4.6 mm in diameter × 250 in length mm. Absorbance was monitored at 214 nm using a Waters 2998 photodiode array detector (Waters Corp., Milford, MA, USA). Peptide peak area was integrated using Waters Empower 2 software with 1 × 10^−9^ mol defined as 1,500,000 units.

### 3.5. Chemical Synthesis of α-CTx TxIA by One-Step Oxidation

Resin-bouned peptides of α-CTx TxIA isomers for one-step oxidation were assembled by solid-phase methodology on an ABI 433A peptide synthesizer using Fmoc chemistry. Resin and regents were prepared using a similar procedure as that used for two-step method. All cysteines were protected with *S*-trityl groups. The linear peptide was removed from a solid support (resin-bounded peptide) by treatment with reagent K. The released peptide was precipitated and washed several times with cold ether. The linear peptide was purified by RP-HPLC using a preparative C_18_ Vydac column with the same linear gradient of above two-step oxidation. Identity of the linear peptide was confirmed by ESI-MS analysis. The linear peptide was lyophilized after purification. Air oxidation folding was carried out in a buffered solution of 0.1 M NH_4_HCO_3_, pH 8.0, containing 1 mM EDTA at room temperature for 72 h. The final peptide concentration was 20 µM. The reaction mix was then quenched by acidification with formic acid of 8% final concentration. The samples were separated by analytical reversed-phase C_18_ HPLC using the above method.

### 3.6. Reduction and Reoxidation of Native α-CTx TxIA

Native and synthetic α-CTx TxIA isomers by the two-step oxidation were reduced, purified by HPLC, and then reoxidized using above air oxidation folding buffer. Reduction was carried out at 37 °C for 45 min. Reducing buffer was 0.1 M Tris, pH 8.5, 1 mM EDTA, and 50 mM DTT. 8% volume formic acid was added to the solution at the end of the reaction. Reduced peptide was purified by HPLC and lyophilized. Folding of fully reduced α-CTx TxIA was performed in 0.1 M NH_4_HCO_3_ (pH 8.0, RT), 1 mM EDTA. The reaction was quenched by acidification with 8% final concentration of formic acid after folding.

### 3.7. RNA Preparation

The cRNAs of rat α3 and β2 nAChR subunits were obtained by *in vitro* transcription using the mMessage mMachine SP6 kit (Ambion, Austin, TX, USA). The cRNAs were purified using the MEGAclearTM kit (Ambion). The concentration of each cRNA was determined by Smart SpecTM plus Spectrophotometer (Bio-Rad). Oocytes of *Xenopus laevis* were prepared and injected with capped RNA (cRNA) to express of rat α3β2 nAChRs.

### 3.8. Voltage Clamp Recording and Data Analysis

Oocytes were harvested and injected with cRNA encoding α3 and β2 nAChR subunits as described previously [17]. Briefly, Oocytes were transferred to the recording chamber (~50 μL in volume) and gravity-perfused at 2 mL/min with ND-96 buffer (96 mM NaCl, 2.0 mM KCl, 1.0 mM MgCl_2_·6H_2_O, 1.8 mM CaCl_2_·2H_2_O, 5 mM HEPES, pH 7.1–7.5) containing 1 μM atropine and 0.1 mg/mL bovine serum albumin (BSA). ACh-gated currents were obtained with a two-electrode voltage-clamp amplifier (Axoclamp 900A, Molecular Devices Corp., Sunnyvale, CA, USA), filter through a 5 Hz low-pass Bessel style filter and digitized at 100 Hz using an Axon Digidata 1440 Data Acquisition System (Molecular Devices Corp.). The oocyte membranes were clamped at a holding potential of −70 mV and data were captured with software pClamp 10.2 (Molecular Devices Corp.). The continuous gravity perfused with standard ND-96 solution and stimulated with 2-s pulses of ACh once every minute. For screening of receptor for toxin concentration 10 μM and lower, once a stable baseline was achieved, added 5 μL of different concentration toxin to the chamber and waited for 5 min, then applied perfusion system, during which 2-s pulses of 100 μM ACh were applied every minute until a constant level of block was achieved. The electrophysiology data were recorded and analyzed using Clampfit 10.2 software (Molecular Devices Corp.). The final results were acquired at least six oocytes. The dose-response data were fit to the equation, %Response = 100/{1 + ([toxin]/IC_50_)n_H_}, Each data point of a dose-response curve represents the average ± S.E. of six to ten oocytes, where n_H_ is the Hill coefficient, by nonlinear regression analysis using GraphPad Prism (GraphPad Software, San Diego, CA, USA).

### 3.9. Circular Dichroism (CD) Spectroscopy

CD spectra were recorded on a JASCO J-810 spectropolarimeter (JASCO International Co., Tokyo, Japan). Spectra were recorded at room temperature under nitrogen atmosphere. Peptides were dissolved in 20 mM ammonium bicarbonate buffer at pH 7. The peptide concentration was determined by quantitative RP-HPLC as above. Spectra were recorded over a 190–260 nm range at 25 °C using an average of 10 scans (scan speed of 10 nm/min). CD data in ellipticity was converted to mean residue ellipticity ([*θ*]R) using the equation: [*θ*]*R* = *θ*/(10 × *C* × *N*p × *l*) where *θ* is the ellipticity in millidegrees, *C* is the peptide molar concentration (M), *l* is the cell path length (cm), and *N*p is the number of peptide residues.

## 4. Conclusions

In this study, we used RP-HPLC and ESI-MS to analyze venom of a *C. textile* cone snail native to Hainan. As we found, *C. textile* venom contains numerous distinct peptides, providing new information on *C. textile* venom complexity and diversity. Screening venom fractions against neuronal α3β2 nAChRs, we identified the most potent blocking component that was identical to α-CTx TxIA, an antagonist of α3β2 nAChRs. We further utilized a chemical method to synthesize three isomers of α-CTx TxIA by two-step oxidation. Bioactivity assays of the three synthesized disulfide isomers of α-CTx TxIA showed that the globular isomer was the most active form. Ribbon isomer was ~80-fold less potent than native globular isomer, while beads isomer showed little activity. The globular and ribbon isomers of α-CTx TxIA had similar retention times in the HPLC profile. Finally we observed that two-step oxidation synthesis was the best way to make the peptide, while one-step synthesis by random oxidation folding was not suitable. We anticipate that further studies of conotoxin disulfide patterns and conotoxin activity would help in elucidating structure-function relationship of these venom peptides.

## Abbreviations

AChBPs: Acetylcholine binding proteins
Acm: Acetamidomethyl
ACN: acetonitrile
CTx: conotoxin
CD: Circular dichroism
EDTA: ethylenediaminetetraacetic acid
ESI-MS: electrospray ionization-mass spectrometry
Fmoc: 9-fluorenylmethyloxycarbonyl
IT-TOF MS: ion trap time-of-flight mass spectrometer
nAChRs: nicotinic acetylcholine receptors
OtBu: tertiary butyl ester
Pbf: 2,2,4,6,7-pentamethyldihydrobenzofuran-5-sulfonyl
RP-HPLC: Reversed-phase high-performance liquid chromatography
*t*Bu: *t*-Butyl
TFA: trifluoroacetic acid
Trt: Trityl
